# Simultaneous optimization of multiple plans within one treatment course with dosimetric pathfinding for temporally feathered radiation therapy

**DOI:** 10.1002/mp.18123

**Published:** 2025-09-10

**Authors:** Julius Arnold, Chengchen Zhu, Gian Guyer, Silvan Mueller, Barbara Knäusl, Olgun Elicin, Marco FM Stampanoni, Peter Manser, Michael K Fix, Jenny Bertholet

**Affiliations:** ^1^ Division of Medical Radiation Physics and Department of Radiation Oncology Inselspital Bern University Hospital and University of Bern Bern Switzerland; ^2^ Department of Radiation Oncology & Christian Doppler Laboratory for Image and Knowledge Driven Precision Radiation Oncology Medical University of Vienna Vienna Austria; ^3^ Department of Physics ETH Zürich Zürich Switzerland; ^4^ Institute of Biomedical Engineering ETH Zürich and PSI Villigen Switzerland

**Keywords:** dynamic trajectory radiotherapy, simultaneous optimization, temporally feathered radiotherapy

## Abstract

**Background:**

Radiotherapy workflows conventionally deliver one treatment plan multiple times throughout the treatment course. Non‐coplanar techniques with beam angle optimization or dosimetrically optimized pathfinding (DOP) exploit additional degrees of freedom to improve spatial conformality of the dose distribution compared to widely used techniques like volumetric‐modulated arc therapy (VMAT). The temporal dimension of dose delivery can be exploited using multiple plans (sub‐plans) within one treatment course. For instance, temporally feathered radiation therapy (TFRT) uses iso‐curative sub‐plans to deliver an alternance of higher and lower doses compared to a single plan to selected organs‐at‐risk (OARs), facilitating the dynamic recovery process of healthy tissues.

**Purpose:**

This study presents a simultaneous optimization framework based on direct aperture optimization with or without DOP to optimize multiple coplanar or non‐coplanar sub‐plans within one treatment course and demonstrates its use for TFRT planning.

**Methods:**

The goal of the framework was to minimize an objective function consisting of weighted upper or lower dose‐volume, generalized equivalent uniform dose, and normal tissue objectives set on the dose distribution of each sub‐plan or the combined total plan. Reference VMAT and dynamic collimator trajectory radiotherapy (colli‐DTRT) single plans were created and used to derive the objectives for TFRT planning. The TFRT high‐to‐low dose modulation was integrated into the objective list and systematically investigated for a digital academic phantom using three variations (“soft”, “medium”, “hard”). Additionally, a “super‐soft” variation used the same objectives for all five sub‐plans (i.e., no high‐to‐low dose modulation). Furthermore, “medium” TFRT sub‐plans with colli‐DTRT were created for three more complex clinically motivated head and neck cases.

**Results:**

For the phantom, the sub‐plans were iso‐curative with target D_98%_ within 1.6% of the reference plans. High‐to‐low OAR dose modulation was achieved with median D_mean_ differences between high and low dose fractions of 2.7% of the prescription dose (soft), 3.3% (medium), and 4.4% (hard) for VMAT. Median OAR D_mean_ differences were 2.8% of the prescription dose (soft), 6.3% (medium), and 6.1% (hard) for colli‐DTRT. The dose distributions of the total plans had higher homogeneity indices (HI = D_98%_/D_2%_) compared to the reference plans. Lower OAR D_mean_ were achieved for the soft, medium, and hard TFRT variation in the total plans compared to the reference plan. However, in the super‐soft variation only five of the 10 feathered OARs showed this reduction.

For the three clinically motivated cases with colli‐DTRT, median OAR D_mean_ differences between the high and low dose fractions were 3.5%, 4.2%, and 7.5% of the prescription dose. The total plans’ dose distributions had higher HIs compared to their respective reference plans and lower or equal D_mean_ for all feathered OARs except for pharynx in one case.

**Conclusion:**

A framework for the simultaneous optimization of multiple coplanar or non‐coplanar sub‐plans within one treatment course was developed. Simultaneous optimization was investigated with a phantom and three clinically motivated cases for TFRT planning. OAR dose modulation in the sub‐plans was achieved while increasing target homogeneity and reducing OAR doses in the dose distribution of the total plans compared to the reference plans.

## 1 INTRODUCTION

Radiation therapy aims to irradiate tumors with a lethal dose while minimizing harm to healthy tissues. In clinical practice, techniques such as intensity‐modulated radiation therapy (IMRT)^1^ and volumetric‐modulated arc therapy (VMAT)^2^ are used to optimize spatial dose distributions, ensuring that the planning target volume (PTV) receives the prescribed dose while minimizing dose to organs‐at‐risks (OARs). Emerging techniques in non‐coplanar radiation therapy^3^, ^4^, ^5^, ^6^, ^7^, ^8^ such as those exploiting dosimetrically optimized pathfinding (DOP)^7^, ^8^, ^9^, ^10^ to find partial non‐coplanar arcs or dynamic gantry‐table‐collimator trajectories, have been investigated to further improve dose conformality to the target. With this, we may be reaching a limit in our capability to optimize spatial dose distribution.

There is growing interest in exploring the temporal dimension of dose delivery using multiple plans (sub‐plans) within one treatment course. Instead of splitting the total dose distribution into equal fractions, the treatment plan is deliberately designed to deliver different spatial dose distributions in different fractions over the treatment course.^11^, ^12^, ^13^, ^14^, ^15^, ^16^ In spatiotemporal fractionation^12^, ^13^ the goal is to maintain fractionation at the normal tissue level with approximately the same (low) dose every day, while hypofractionating different parts of the tumor on different days based on biological effective dose (BED) optimization of the total plan. In temporally feathered radiotherapy (TFRT)^14^, ^15^ the goal is to deliver the same homogenous dose to the tumor at each fraction (the sub‐plans are iso‐curative) but to modulate the dose to the OARs. Selected OARs each get a higher dose than in a reference single plan in one sub‐plan and lower doses in the remaining sub‐plans to exploit the dynamic recovery process of healthy tissues, thereby reducing modeled toxicity. Allowing for multiple sub‐plans instead of one single plan also has advantages from a pure physical dose optimization point of view by increasing the optimizer's degrees of freedom (DOF) when the same number of IMRT fields or dynamic arcs are used for each sub‐plan as for a single plan.^16^ However, defining different beam configurations and hence, different dose distributions, for a single treatment course poses a complex optimization problem.

Several previous approaches tried to address these challenges. Simultaneous optimization of multiple plans for spatiotemporal fractionation was achieved using BED‐based optimization of fluence maps, but optimized only the summed BED without control of sub‐plan dose distribution.^11^, ^13^ Additionally, deliverability was not ensured. Rossi et al.^16^ proposed “per‐fraction planning” to sequentially generate one sub‐plan for each new fraction by adding dose to the already generated fraction sub‐plans for treatments on a robotic linac. Their approach leveraged beam angle optimization to reduce OAR dose in the total plan compared to a single plan approach but biological effects were neglected and the optimization of all sub‐plans was not simultaneous.^17^ For TFRT, sub‐plans for clinical cases and a first in‐human study were created individually in a clinical treatment planning system.^15^, ^18^ However, this manual and iterative process is cumbersome and may yield a sub‐optimal solution. A more comprehensive optimization strategy is needed to leverage the available degrees of freedom with several sub‐plans, especially for TFRT.

In this study, we developed simultaneous optimization of multiple sub‐plans within one treatment course including optional DOP for C‐arm linacs. The developed simultaneous optimization framework optimizes deliverable sub‐plans, using objectives set on the dose distribution of each sub‐plan and of the total plan. The use of the framework was demonstrated for TFRT planning on a digital academic phantom as well as three clinically motivated head and neck cases.

## 2 MATERIAL & METHODS

An in‐house optimizer using Monte Carlo calculated beamlet doses and direct aperture optimization (DAO)^19^, ^20^ (Section 2.1.) was extended to enable the simultaneous optimization of multiple sub‐plans (Section 2.2). This simultaneous optimization workflow was also implemented for DOP to exploit different, but dependent, non‐coplanar beam directions in different sub‐plans (Section 2.3). A digital academic phantom was designed and used to systematically investigate the behavior of simultaneous optimization with and without DOP, while clinically motivated cases were considered to evaluate the performance of simultaneous optimization in complex realistic cases (Section 2.4).

### 2.1 In‐house treatment planning process

The treatment planning process has been described in detail elsewhere for multiple treatment techniques.^8^, ^9^, ^10^, ^19^, ^20^, ^21^ In short, the CT is imported into a research version of the commercial treatment planning system Eclipse 15.6 (Varian, a Siemens Healthineers Company, Erlangen, Germany), in which the structures are contoured.^9^, ^19^, ^20^, ^22^ The field set‐up can be either manually defined or dosimetrically optimized (Section 2.3). Dynamic paths are discretized into a series of control points. Beamlet doses for each control point are calculated using the Swiss–Monte–Carlo‐plan (SMCP)^23^ framework with validated pre‐simulated phase‐space located at the treatment head exit plane for a Varian TrueBeam. The intensity modulation is subsequently optimized with a DAO algorithm.^19^, ^20^ This algorithm combines column generation and simulated annealing with an additional branch feature to iteratively add the aperture leading to the lowest objective function value to the aperture pool.^19^ The goal of the optimization is to minimize an objective function consisting of upper and lower dose‐volume objectives, generalized uniform dose objectives (gEUD), and normal tissue objectives.^19^, ^24^, ^25^, ^26^

Final dose calculation for photon plans is performed in SMCP using VMC++^27^ simulating the plan‐specific part of the linac head including secondary collimator jaws and multileaf collimator, to calculate dose deposition in the phantom or patient, accounting for the dynamic motion of all linac components. Monitor unit (MU) weights are then re‐optimized using a limited‐Broyden–Fletcher–Goldfard–Shanno (L‐BFGS) algorithm to mitigate discrepancies between beamlet‐based and final dose calculation.

### 2.2 Simultaneous optimization workflow

The simultaneous optimization workflow was implemented to simultaneously handle P sub‐plans (*i* = 1, …, P) within this treatment planning process, each with a set of apertures $A_{i}$, a dose distribution $d_{i}$ for a given number of fractions $n_{i}$, a set of objectives $O_{i}$, and an associated objective function $F_{i}\left(d_{i},D_{tot}\right)$. The objective function $F_{i}$ of each sub‐plan can consist of objectives defined on the dose distribution of an individual sub‐plan ($f(d_{i})$) and on the dose distribution of the total plan ($f(D_{tot})$), where the total dose distribution $D_{tot}$, is the sum of all the sub‐plans’ physical dose distributions $d_{i}$ (Equation 1).

(1) $$D_{tot}=\sum_{i=1}^{P}n_{i}d_{i}$$

This allows to include the intentional OAR dose modulation into the lists of objectives for TFRT plan generation (Section 2.4).

**FIGURE 1:**
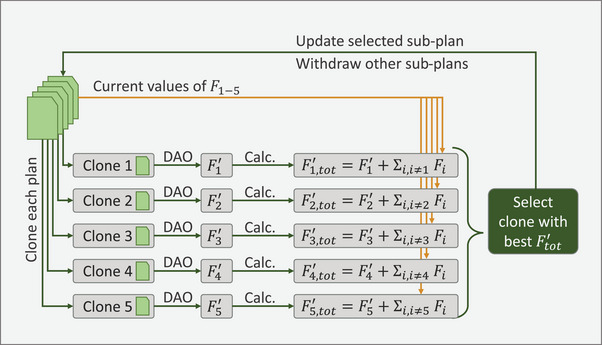
Simultaneous optimization workflow to distribute the DAO iterations between the sub‐plans. Here, illustrated for five sub‐plans (*p* = 5).

The simultaneous optimization is embedded in the iterative DAO structure, where one aperture is added to the aperture pool of one sub‐plan in each DAO step as illustrated in Figure 1.

One aperture is added to one sub‐plan per iteration. To determine to which sub‐plan the aperture is added, a copy of each sub‐plan (clone) is created from the current state of the optimization. Then, on each clone, one DAO step is performed leading to a new value for the objective function, ${F_{j}}^{\prime }$ for clone j. The total objective function is calculated for each clone according to Equation 2, where $F_{j,\;\mathrm{tot}}^{\prime }$ is the total objective function value of clone j, $F_{i}$ is the objective function value of sub‐plan i in the current optimization state.

(2) $${F^{\prime }}_{j,\mathrm{tot}}={F^{\prime }}_{j}+\sum_{i=1,i\neq j}^{p}F_{i}$$

The clone leading to the lowest total objective function value is accepted, and the corresponding sub‐plan is updated. The other clones are deleted, and the next iteration is launched. The simultaneous optimization workflow is terminated when all control points have been assigned the desired number of apertures (one aperture per control point for dynamic arcs).

After final dose calculation for each sub‐plan, the MU‐weight re‐optimization is performed using the simultaneous optimization workflow with L‐BFGS MU‐weight optimizations instead of DAO steps until the total objective function does not further decrease.

The computation time (CPU time) of this simultaneous workflow scales linearly with the number of sub‐plan.

### 2.3 Dosimetrically optimized pathfinding

DOP can be used for dynamic trajectory radiotherapy by considering all collision‐free beam directions for the included dynamic axes (gantry angle, table rotation) using an in‐house collision prediction tool.^8^, ^9^, ^10^, ^28^ The DOP follows the structure of the DAO with apertures being selected from the collision‐free beam directions according to the rules of the respective treatment technique.^9^, ^10^ The simultaneous optimization workflow is readily applicable to the DOP part of the treatment planning process.^29^

In this study, we considered the treatment technique colli‐DTRT, which uses non‐coplanar partial arcs and dynamic collimator rotation to maintain the collimator aligned to the patient's superior‐inferior axis.^8^, ^9^ Collision‐free beam directions were sampled from a 10°× 10° gantry‐table angle grid using an isocentric set‐up; those entering through the end of the CT were discarded. Beamlet doses were calculated using SMCP for the remaining beam directions with a voxel size of 5 × 5 × 5 mm^3^. The DOP was constrained to consider paths at a fixed table angle for a user‐specified maximum number of arcs and the maximum total gantry angle range as described in detail in Zhu et al.^9^

After DOP, the dynamic arcs were resampled with a control point spacing of 5°, and beamlet doses were re‐calculated for a finer voxel size of 2.5 × 2.5 × 2.5 mm^3^ using SMCP (this control point spacing and voxel size were applied to the VMAT plans of the present study as well). The intensity modulation of the resampled arcs was re‐optimized with the DAO, followed by final dose calculation and MU weight re‐optimization as described in Section 2.2.

For the simultaneous optimization of colli‐DTRT plans, in each step of the treatment planning process the simultaneous optimization workflow was used with DOP, DAO, and L‐BFGS MU‐weight optimizations. During the DOP each sub‐plan was subject to the same maximum gantry angle range and maximum number of arcs per sub‐plan as the corresponding reference plan.

For DOP, CPU time scales linearly with the number of sub‐plans but memory usage is unchanged as the same search‐space is considered for all sub‐plans. However, for simultaneous optimization of the intensity modulation along the paths and L‐BFGS MU‐weight optimization, memory use increases depending on how many beamlets are used by several sub‐plans.

### 2.4 Application of the simultaneous optimization to TFRT planning

#### 2.4.1 Definition of objectives for TFRT

In the present study, we used five sub‐plans as in previous TFRT studies.^14^, ^15^ We took a practical approach to obtain objectives for TFRT planning, similar to Parsai et al.^15^ based on the premise that OAR toxicity is related to the high‐to‐low dose modulation. For each case, a reference single plan was created first using the in‐house optimizer. The objectives of this reference plan were used to derive objectives for TFRT planning. The total dose objectives ($f(D_{tot})$) were the same as for the reference plan. For the target volume, normal tissue, and the non‐feathered OARs, the same objectives were applied to each sub‐plan ($f(d_{i})$). However, the objected OAR doses in the sub‐plans’ objectives were varied to achieve the TFRT feathering (high‐to‐low dose modulation) for five OARs. The dose difference factor $d_{\mathrm{diff}}=d_{h}\;-d_{l}$ was introduced as the difference of the objected high dose ($d_{h}$) and the objected low dose value ($d_{l}$). Additionally, we chose to satisfy $\frac{d_{h}+4d_{l}}{5}=d_{\mathrm{re}f}$, where $d_{\mathrm{re}f}$ is the objected dose in the reference plan, in the objectives to focus on the high‐to‐low dose modulation without intentionally enforcing a reduction of the total physical dose. Given the objected dose in the reference plan and a selected dose difference factor, the objected high dose was calculated as $d_{h}=d_{\mathrm{ref}}+\frac{4}{5}d_{\mathrm{diff}}$, while the objected low dose was calculated as $d_{l}=d_{\mathrm{ref}}+\frac{1}{5}d_{\mathrm{diff}}$.

### 2.5 Phantom case

To systematically investigate the performance of simultaneous optimization, a digital phantom case was designed which consisted of a cylindrical body with a spheroid PTV placed slightly off‐center. Five spheroid OARs were arranged asymmetrically in a pentagonal distribution around the PTV with small variations in their distance to the PTV and their longitudinal position (OARs 1–5). Details of the phantom design can be found in Figure S1.^30^

Two reference single plans were created for a prescription of 50 Gy in 2 Gy per fraction to 95% of the PTV. One plan used a single full VMAT arc (VMAT_ref_) and one used DOP to create a colli‐DTRT plan for 360° total gantry angle range with a maximum of seven partial arcs (colli‐DTRT_ref_).

For each technique, four TFRT variations were created for $d_{\mathrm{diff}}$ of 30% of the prescription dose (“hard” TFRT), 20% (“medium” TFRT), 10% (“soft” TFRT), and 0% (“super‐soft” TFRT). The soft, medium, and hard variations were chosen to systematically investigate the simultaneous optimization framework for an increasing demand on the high‐to‐low dose modulation, while the super‐soft variation was chosen to investigate only the impact of the increased DOF provided by the multiple sub‐plans. VMAT and colli‐DTRT were compared to evaluate the impact of adding simultaneous DOP on the high‐to‐low dose modulation.

### 2.6 Clinically motivated cases

For evaluation in more complex and realistic situations, TFRT sub‐plans were created using colli‐DTRT and the medium TFRT variation ($d_{\mathrm{diff}}$ of 20% of the prescription dose) for the elective nodal volume of three oropharyngeal carcinoma cases with a prescription of 50 Gy to 95% of the PTV, delivered in 25 fractions.

The DOP settings to create the reference colli‐DTRT plans (colli‐DTRT_ref_) were chosen based on PTV size as shown in Table 1. If PTV required a field larger than 15 cm, field splitting with x‐jaws was applied as described in Zhu et al.^9^

**TABLE 1:** Description of the three clinically motivated cases and settings used for DOP.

	Description	PTV volume [cm^3^]	Total gantry angle range	Max number of arcs	Field splitting
Case 1	Local elective nodal volume	85.79	720°	6	No
Case 2	Unilateral elective nodal volume	303.51	1080°	8	Yes
Case 3	Bilateral elective nodal volume	325.96	1440°	10	Yes

Pharynx, larynx, oral cavity, left parotid gland, and right parotid gland were the selected OARs to feather because of their importance for radiation‐induced xerostomia and dysphagia.

### 2.7 Evaluation

The dose distributions of the sub‐plans, the total plans and the reference plans were compared based on D_mean_ for the feathered OARs and PTV homogeneity indices $\left(\mathrm{HI}=D_{98\%}/D_{2\%}\right)$ as well as relevant dosimetric endpoints for non‐feathered OARs.^15^, ^18^ To assess the TFRT modulation, the median and range of the differences in OAR D_mean_ between the high dose fraction and the low dose fractions were reported across the feathered OARs.^18^ All sub‐plans for one of the clinically motivated cases were delivered on a TrueBeam in developer mode. Delivery logfiles were analyzed to quantify mechanical accuracy.

## 3 RESULTS

### 3.1 Phantom case

**FIGURE 2:**
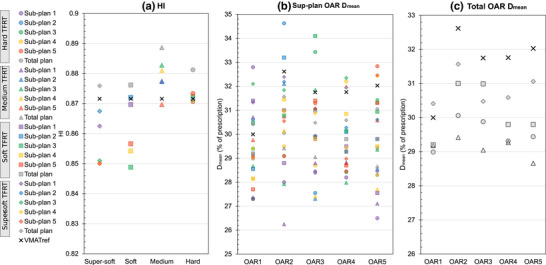
HIs (a) and OAR D_mean_ (b, c) of all (sub‐)plans for all TFRT variations using VMAT and the phantom case. TFRT variations are represented by circles (hard), triangles (medium), squares (soft), and diamonds (super‐soft). Sub‐plans 1–5 are sequentially colored in purple, blue, green, yellow, and red, with total plans in gray. VMAT_ref_ plan values are indicated by black crosses.

The VMAT sub‐plans generated using the developed framework had different aperture shapes and weights, leading to different dose distributions in each sub‐plan. The sub‐plans were iso‐curative with PTV D_98%_ within −0.5% and +1.6% of the reference plan. The HIs of the VMAT_ref_ plan, all sub‐plans, and total plans for all TFRT variations are presented in Figure 2a. The HIs of the sub‐plans were within ± 0.2% of the HI in the VMAT_ref_ plan for the hard and within ± 1.3% for the medium TFRT variation. The variations reached −2.5% for the super‐soft and the soft TFRT variations. However, the HI was higher in the dose distribution of the total plan than for the VMAT_ref_ plan for all TFRT variations.

D_mean_ for the five OARs in all (sub‐)plans and TFRT variations are shown in Figure 2b,c. The high‐to‐low dose modulation pattern is generally visible in the case of the soft, medium, and hard TFRT variation. The median (range) D_mean_ difference between the high and low dose fractions over all feathered OARs was 1.7% (0.7%–2.1%) of the prescription dose in the super‐soft, 2.7% (1.2%–3.9%) in the soft, 3.3% (1.9%–3.5%) in the medium, and 4.4% (3.6%–5.7%) in the hard TFRT variation. The median (range) differences between the OAR D_mean_ of the total plan and the VMAT_ref_ plan was ‐1.1% (−1.3% to 0.4%) of the prescription dose in the super‐soft, −1.6% (−2.2% to ‐0.8%) in the soft, −2.7% (−3.4% to −0.8%) in the medium, and −2.5% (−2.6% to −1.0%) in the hard variations. There was a reduction of the total OAR D_mean_ for all OARs and all TFRT variations compared to the VMAT_ref_ plan, except for OAR1 in the super‐soft TFRT variation. The objective function value evaluated on the dose distribution of the VMAT_ref_ plan was 0.061, it was 0.058 when evaluated on the dose distribution of the total super‐soft variation. The DAO optimization took 192 s (CPU time) for the VMAT_ref_ plan and 1518 s for the super‐soft variation.

The simultaneous DOP resulted in different colli‐DTRT arc set‐ups for the different sub‐plans. The selected arcs are shown in Figure S2 with the corresponding dose distributions for the medium TFRT variation. OAR D_mean_ were lower in all (sub‐)plans using colli‐DTRT compared to those using VMAT.

**FIGURE 3:**
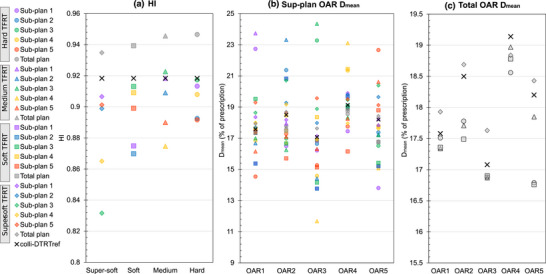
HIs (a) and OAR D_mean_ (b, c) of all (sub‐)plans for all TFRT variations using colli‐DTRT and the phantom case. TFRT variations are represented by circles (hard), triangles (medium), squares (soft), and diamonds (super‐soft). Sub‐plans 1–5 are sequentially colored in purple, blue, green, yellow, and red, with total plans in gray. colli‐DTRT_ref_ plan values are indicated by black crosses.

The sub‐plans were iso‐curative with PTV D_98%_ within −0.2% and +1.2% of the reference plan. The HIs and OAR D_mean_ of all (sub‐)plans are presented in Figure 3. The HIs of all sub‐plans for all TFRT variations were below the HI of the colli‐DTRT_ref_ plan by up to 9.4% except for sub‐plan three in the medium TFRT variation where it increased by 0.5%. The HIs of all total plans increased compared to the colli‐DTRT_ref_ plan.

Figure 3b shows the resulting OAR D_mean_, where the high‐to‐low dose modulation was observed for all OARs in the soft, medium and hard TFRT variations with median (range) OAR D_mean_ differences of 2.8% (2.1%–9.4%) of the prescription dose in the soft, 6.3% (3.6%–9.5%) in the medium, and 6.1% (3.0%–7.6%) in the hard TFRT variations. D_mean_ in the total plan decreased for all OARs compared to the colli‐DTRT_ref_ plan, with a median (range) OAR D_mean_ reduction of 0.4% (0.2%–1.4%) of the prescription dose in the soft, 0.2% (0.2%–0.8%) in the medium, and 0.6% (0.1%–1.4%) in the hard TFRT variations. The super‐soft TFRT variation showed random modulation of the OAR D_mean_ compared to the colli‐DTRT_ref_ plan, with median (range) D_mean_ differences of 1.8% (0.7%–1.9%) of the prescription dose. D_mean_ in the total plan increased for four OARs compared to the colli‐DTRT_ref_ plan, leading to a median OAR D_mean_ increase of 0.2% of the prescription dose. However, the objective function evaluated on the dose distribution of the total super‐soft variation was 0.045, whereas it was 0.064 for colli‐DTRT_ref_ plan. The DAO optimization took 400 s (CPU time) for the colli‐DTRT_ref_ and 1954 s for the super‐soft variation.

### 3.2 Clinically motivated cases

**FIGURE 4:**
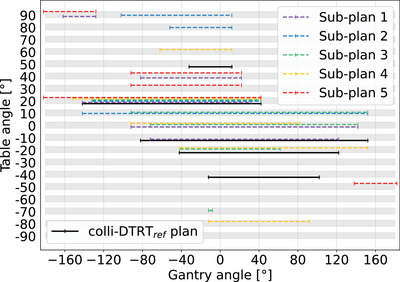
Selected arcs for all sub‐plans and the colli‐DTRT_ref_ plan for Case 3. Animation of the selection process is shown in Video S1.

For all cases, the simultaneous DOP selected different arc set‐ups for the sub‐plans, which in total covered a larger portion of the 4π‐space than the arcs in the colli‐DTRT_ref_ plan as shown in Figure 4 for Case 3. The iterative selection process of these apertures is presented in Video S1. The selected arcs for the other two cases are shown in Figures S3 and S4.

Table 2 summarizes the D_mean_ of the feathered OARs and the HIs in the TFRT and reference colli‐DTRT plans for the three clinically motivated cases.

**TABLE 2:** Summary of the OAR D_mean_ and the HIs of all (sub‐)plans of the three clinical cases. The best D_mean_ and HI between the total TFRT plan and the reference plan are underlined.

	Case 1			Case 2			Case 3		
	Sub‐plans median (range)	Total plan	colli‐DTRT_ref_ plan	Sub‐plans median (range)	Total plan	colli‐DTRT_ref_ plan	Sub‐plans median (range)	Total plan	colli‐DTRT_ref_ plan
OAR D_mean_ (% of prescription)									
Pharynx	47.0 (45.0–50.0)	46.9	47.5	52.3 (52.0–56.6)	52.7	52.2	89.1 (88.5–95.1)	89.2	89.3
Larynx	17.8 (17.3–19.4)	18.1	18.8	32.0 (31.2–39.9)	33.0	34.1	39.2 (38.1–52.8)	41.0	41.1
Parotid R	14.7 (13.2–22.9)	15.9	15.9	6.8 (5.0–6.9)	5.4	6.1	23.7 (22.6–30.2)	23.6	24.5
Parotid L	2.7 (1.6–3.5)	2.6	3.2	60.6 (60.5–61.6)	60.2	61.7	48.9 (48.1–57.5)	49.9	50.3
Oral Cavity	45.8 (43.2–56.0)	46.8	46.8	38.7 (37.3–43.8)	38.9	38.9	42.4 (41.8–50.1)	43.6	43.9
PTV HI = D_98%_ / D_2%_									
	0.92 (0.91 – 0.93)	0.94	0.93	0.90 (0.90 – 0.91)	0.93	0.92	0.89 (0.88 – 0.89)	0.91	0.90

The sub‐plans were iso‐curative with PTV D_98%_ within ± 0.5% for all cases. The HIs of the total plans increased compared to the colli‐DTRT_ref_ plans in all cases. Detailed values of the HIs and the D_mean_ of feathered OARs are presented in Figures S3–S5 for all three cases. High‐to‐low dose modulation was achieved for all OARs. The median (range) D_mean_ differences between the high and low dose fractions of the feathered OARs were 3.5% (1.0%–11.1%) of the prescription dose for Case 1, 4.2% (1.0%–8.2%) for Case 2, and 7.5% (6.2%–4.2%) for Case 3.

In general, the dose to the non‐feathered OARs in the sub‐plans and the total plans were close to the reference plan. All mandatory clinical goals ($D_{0.03cm^{3}}$ for the brainstem [54 Gy] and its planning at risk [PRV] [54 Gy] and for the spinal cord [45 Gy] and its PRV [48 Gy]) were respected. Detailed dose endpoints for the non‐feathered OARs can be seen in Table S1. The overall median (range) D_mean_ differences between the high and low dose fractions of the feathered OARs were 6.2% (1%–14.2%) of the prescription dose. The median decrease in D_mean_ for the total plan compared to the reference plan for the feathered OARs was 0.4% of the prescription dose.

**FIGURE 5:**
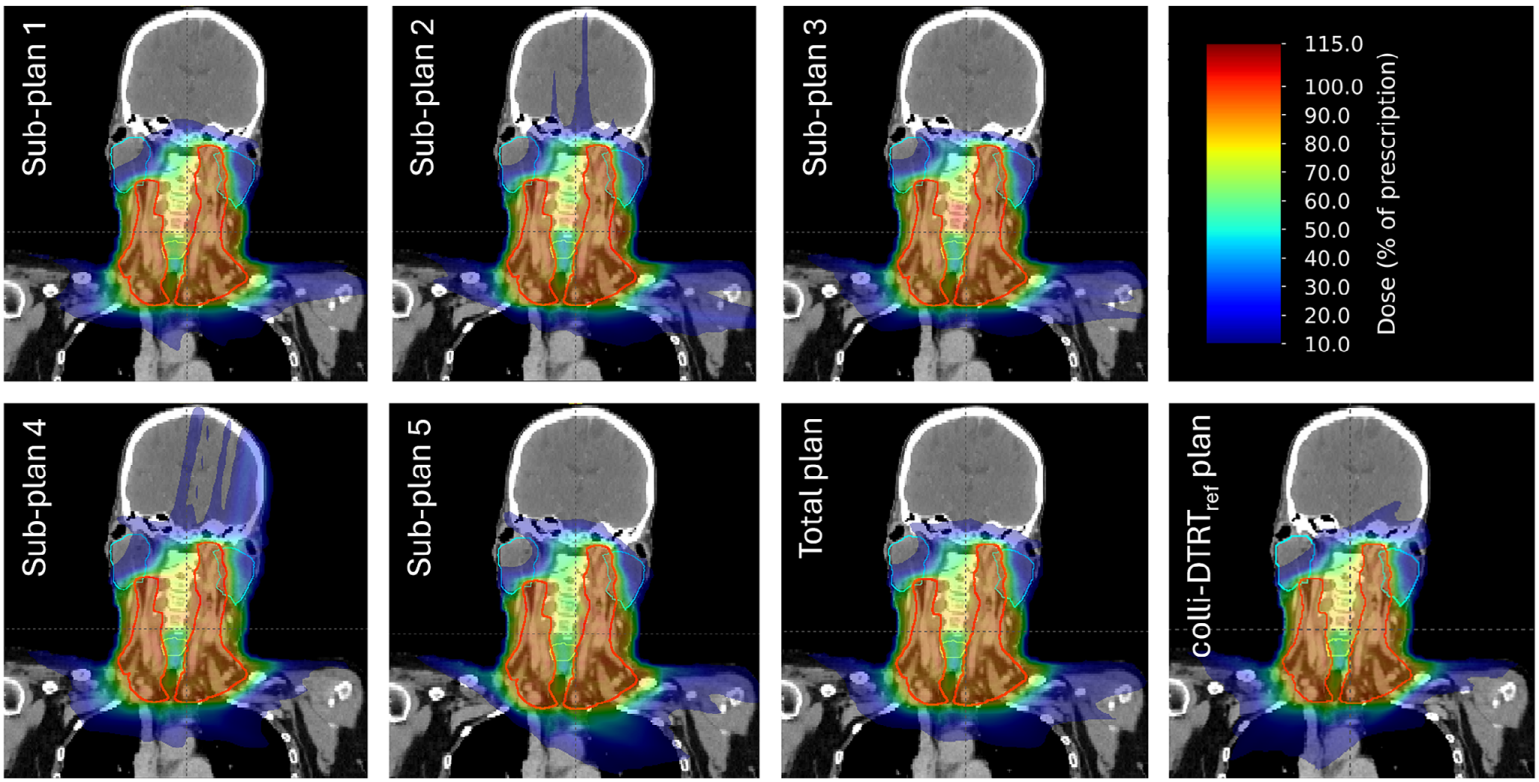
Dose distribution for the central coronal slices of Case 3. PTV contoured in red, pharynx in yellow, and parotid glands in cyan.

**FIGURE 6:**
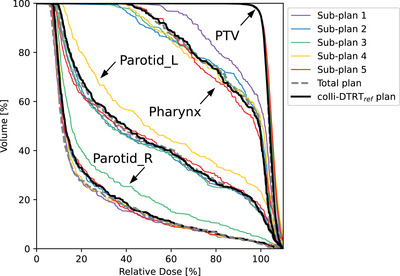
Dose volume histograms of all (sub‐)plans for the PTV, pharynx, and both parotid glands of Case 3.

For Case 3, coronal dose distributions of each sub‐plan, the total plan, and the colli‐DTRT_ref_ plan are shown in Figure 5. Different dose distributions for the different sub‐plans led to a more conformal dose distribution in the total plan compared to the colli‐DTRT_ref_ plan, which can also be observed in the corresponding dose volume histograms for the PTV and three of the feathered OARs in Figure 6.

All sub‐plans for Case 3 were delivered successfully on a TrueBeam in developer mode. Logfiles analysis revealed root‐mean‐square difference between expected and actual values for the following components: 0.025° (gantry angle), 0.024° (collimator angle), 0.026 mm (all moving MLC mean root‐mean‐square difference), and 0.010 for the monitor units.

## 4 DISCUSSION

A simultaneous optimization framework was developed for the optimization of multiple deliverable sub‐plans within one radiotherapy treatment course. The framework is compatible with DOP to exploit different beam directions for the different sub‐plans. Optimization objectives can be defined on the dose distribution of each sub‐plan and of the total plan.

The framework was systematically investigated for the generation of TFRT (sub‐)plans for a digital academic phantom using colli‐DTRT and VMAT. High‐to‐low dose modulation was achieved for the selected feathered OARs across the sub‐plans, while the OAR D_mean_ in the dose distribution of the total plans were lower than in the reference plans for most of the selected OARs. Additionally, all total plans showed increased target homogeneity compared to the reference plans.

Increasing the dose difference factor did not always lead to higher OAR D_mean_ in the high dose fractions and lower OAR D_mean_ in the low dose fractions for each of the selected OARs. However, it did yield a higher median OAR D_mean_ difference between high and low dose fractions. This behavior was likely due to the non‐convex nature of the multi‐criteria optimization problem, particularly in the colli‐DTRT plans, where the search space is larger than for VMAT.

To investigate the dosimetric benefit of increased DOF by optimizing five sub‐plans instead of one, super‐soft variations were investigated with no intentional high‐to‐low dose modulation. For these plans, the HIs of the total dose distributions were also higher than in their corresponding reference plan. However, the OAR dose reduction was not as consistent as in the other TFRT variations with half of the OARs having a higher D_mean_, in the dose distribution of the total plans than in the reference plans. Nevertheless, the objective function evaluated on the dose distributions of the super‐soft TFRT variations were lower than for the corresponding reference plans, indicating that the increased DOF did, as expected, result in a mathematically more optimal solution.

DOP for colli‐DTRT also represents an increase in DOF compared to VMAT and resulted in greater high‐to‐low dose modulation with TFRT planning. The D_mean_ reduction in the total plan compared to the single reference plan was smaller in colli‐DTRT compared to VMAT. This might be because the OAR doses were already substantially lower in the reference colli‐DTRT plan compared to VMAT plan with little added benefit of having multiple plans on the total dose distributions.

In addition to the systematic investigation on a phantom, the framework was also used for TFRT planning on three clinically motivated head and neck cases with a realistic, complex anatomy. The full prescription was 70 Gy to the primary tumor (boost). However, for the purpose of this technical study, we only planned the first phase (50 Gy to elective nodal volume) for both the reference single and the TFRT plan. The target volume for Case 1 resembles a typical high dose volume for head and neck treatments; hence, it is expected that TFRT advantages would also extend to the boost phases. Nevertheless, it is imperative to conduct a more extensive planning study to the full prescription dose with sequential or simultaneous integrated boost in larger cohorts to draw clinically meaningful conclusions.

In previous studies on TFRT for head and neck cancer, each sub‐plan was independently optimized in a commercial treatment planning system which is cumbersome and likely to yield a sub‐optimal solution.^15^, ^18^ Parsai et al.^18^ investigated the feasibility of TFRT in clinical practice for four squamous cell carcinoma patients treated to a prescription of 35 × 2 Gy to the primary tumor using TFRT VMAT sub‐plans. Across the 20 feathered OARs of the four patients, D_mean_ differences between the total plan and the reference IMRT plan ranged from ‐8.0% to +4.1% of the prescription dose, with an increase in total dose for seven OARs. In contrast, across the 15 feathered OARs for the three clinically motivated cases in this study, a D_mean_ reduction in the range of −1.5% and 0% was observed in the total plan compared to the reference plan for 14 OARs, while a small increase of +0.5% was observed for the pharynx D_mean_ in the second case. Furthermore, Parsai et al.^18^ reported OAR D_mean_ differences between the high and low dose fractions with a median (range) of 1.5% (0.3%–3.3%) of the prescription dose. In comparison, D_mean_ differences with a median of 6.2% (1.0%–14.2%) of the prescription dose were achieved in this study over the three cases. The concept of TFRT is based on the hypothesis that OAR toxicity is directly related to the high‐to‐low dose modulation. Hence, greater differences, as obtained in our study with simultaneous optimization, are expected to result in a greater OAR sparing compared to the differences obtained with manual planning. Importantly, feathering did not cause critically higher doses to the non‐feathered OARs in the sub‐plans or in the total plans. Mandatory dose limits to the brainstem and spinal cord were respected in all sub‐plans and total plans. It should be noted that the optimization is currently performed on the physical dose and therefore it remains an indirect way to optimize biological effects. The framework should be further extended to include explicitly formulated objectives for the dynamic normal tissue complication probability (NTCP) model^14^ and BED‐based optimization^12^, ^13^ For this, new objectives need to be included that incorporate biological parameters (α, β, μ, and α/β) for the different structures, the sub‐plans dose distribution, the number of fraction per sub‐plan, and the time between fractions to calculate the total plan's dynamic NTCP and BED.

Simultaneous optimization of multiple sub‐plans is a feature of spatiotemporal fractionation where cumulative BED is optimized based on a fluence map optimization, but without control on the dose distribution of the individual sub‐plans.^12^ More recently, a constrained approach to spatiotemporal fractionation was proposed that enables to obtain more homogeneous target dose distributions in each sub‐plan for multiple brain metastases.^13^ However, OAR and normal tissue doses were only optimized for the cumulative BED (i.e., in the total plan), whereas our framework combines individual sub‐plan and total plan objectives for both target and OARs. In addition, no deliverable plans were generated for spatiotemporal fractionation so far.^11^, ^13^ Deliverability and dosimetric accuracy for plans created using our in‐house optimization framework was shown extensively in previous studies.^4^, ^5^, ^9^, ^10^, ^20^, ^31^, ^32^, ^33^ Here, all sub‐plans for Case 3 were successfully delivered with high mechanical accuracy (based on log‐file analysis).

Our framework is the first to propose simultaneous optimization using standard upper and lower dose‐volume, gEUD, and normal tissue objectives that can be set for each sub‐plan as well as the total plan. This approach increases flexibility and was key to reach high‐to‐low dose modulation for feathered OARs with iso‐curative sub‐plans. Nevertheless, selecting and adjusting objectives is a complex task that scales up with the number of sub‐plans. This may also partly explain the limited benefit of the super‐soft TFRT variation as discussed above. An automated treatment planning strategy should be implemented to fully exploit the potential of simultaneous optimization. Automated planning was used in per‐fraction planning for a robotic linac, but there the sub‐plans were optimized sequentially.^16^, ^34^

Optimization time scaled linearly with the number of sub‐plans, which is in line with other approaches.^12^, ^13^, ^16^ Memory usage also scaled approximately linearly with the number of sub‐plans when using colli‐DTRT because of the diverse beam directions. However, for VMAT, memory usage did not notably increase because all sub‐plans used the same beamlets. The number of optimization iterations needed per sub‐plan increased by a factor 1.2 on average compared to optimization of a single plan. Delivery time is equivalent for TFRT as for reference single plans in this study because the same total gantry angle range was used for each sub‐plan as for the corresponding reference plan.

Delivering multiple sub‐plans within one treatment course represents a practical challenge in terms of time constraint, quality assurance, and workflow. Nevertheless, Parsai et al.^18^ showed that this was feasible within a clinical workflow with 8–15 days between CT simulation and radiation start with manual planning. Simultaneous optimization is expected to substantially reduce the burden on treatment planning. Still, plan specific QA scales with the number of sub‐plans. In addition, care must be taken to deliver the sub‐plans in the right sequence. For this, the use of five sub‐plans is a practical choice since the same sub‐plan is always delivered on the same day of the week, but this would have to be adjusted in case of missed treatment. Addressing these challenges is beyond the scope of the present study, but is essential to clinical implementation.

In the present study, we used five sub‐plans for TFRT planning. However, the developed framework allows for an arbitrary number of sub‐plans, each with an arbitrary prescription dose and arbitrary number of fractions, meaning that any number of sub‐plans can be optimized for TFRT and that other applications can also be envisaged. For example, in the case of sequential boost treatments, the different phases are generally optimized individually and may have to be adjusted iteratively to obtain an acceptable total dose distribution. Certain treatment planning systems allow to optimize the boost phase based on the total dose using the first phase as a “base dose”.^26^ However, this may result in an inhomogeneous dose distribution for the boost phase because there are no objectives applying only to the boost dose distribution. Our framework enables the simultaneous optimization of multiple phases with the possibility to control the dose distribution of the individual phases as well as the total dose distribution. Our framework could also be used to simultaneously optimized plans for multiple targets (e.g., bilateral breast irradiation, multiple metastases) by ensuring adequate target coverage in each (sub‐)plan while keeping better control on the dose to common OARs. Finally, for adaptive radiation therapy, where the adapted plans are generally optimized as a new plan without considering the already delivered dose, our framework could be used to optimize the adapted plan accounting for the already delivered dose.

## 5 CONCLUSION

Simultaneous optimization of multiple sub‐plans within one treatment course was successfully developed, including simultaneous DAO as well as an optional simultaneous DOP. The developed framework uses standard optimization objectives applicable to the sub‐plans and the total plan. The framework was used to produce TFRT sub‐plans for a phantom with different high‐to‐low dose modulations using VMAT or colli‐DTRT and for three clinically motivated cases using colli‐DTRT. High‐to‐low dose modulation was achieved. The total plans had an increased target homogeneity and overall reduced OAR dose compared to their respective single reference plans.

## CONFLICT OF INTEREST STATEMENT

In his role as deputy editor for Medical Physics, author M.K. Fix was blinded to the review process and had no role in decisions pertaining to this manuscript.

## Supporting information



Supporting Information

Supporting information

Supporting information

Supporting information

Supporting information

Supporting information

Supporting information
