# Supplementary material for: Simultaneous optimization of multiple plans within one treatment course with dosimetric pathfinding for temporally feathered radiation therapy
Source: Med Phys. 2025 Sep 10;52(9):e18123. doi: 10.1002/mp.18123 (PMC12421372; doi:10.1002/mp.18123)
Supplement: Supplementary file 1 — Supporting Information [file MP-52-0-s004.pdf]

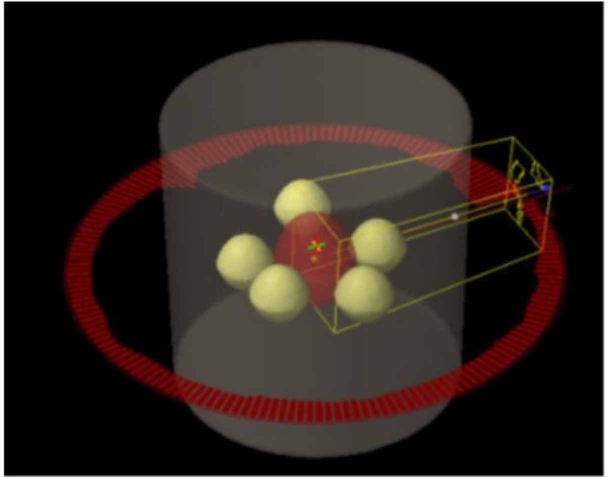

Structure	Dimensions[cm]	Center of mass position [cm] x	y	z	Distance to PTV [cm]
Body	25.0⌀	0	0	0	
PTV	7.0⌀, axial<br>8.6 SI	1.6	−0.9	0	
OAR1	5.0⌀	−2.0	−5.6	−1.2	0.2
OAR2	5.0⌀	5.0	−5.7	0.4	−0.1
OAR3	5.0⌀	7.5	0.7	−0.3	0.1
OAR4	5.0⌀	1.6	5.0	−0.7	0.0
OAR5	5.0⌀	−3.9	0.6	1.3	0.0
