# Supplementary figures and images for: Simultaneous optimization of multiple plans within one treatment course with dosimetric pathfinding for temporally feathered radiation therapy
Source: Med Phys. 2025 Sep 10;52(9):e18123. doi: 10.1002/mp.18123 (PMC12421372; doi:10.1002/mp.18123)

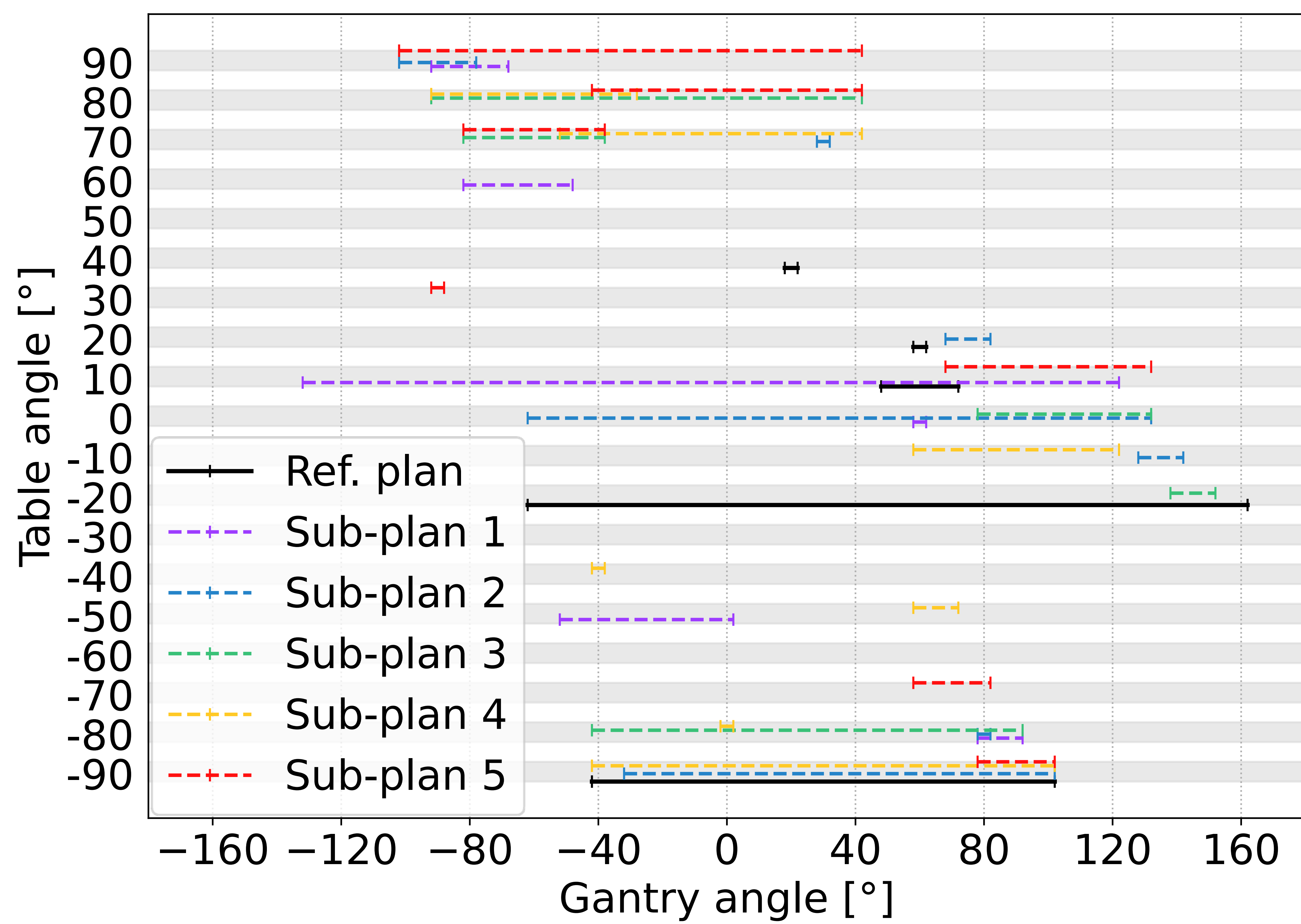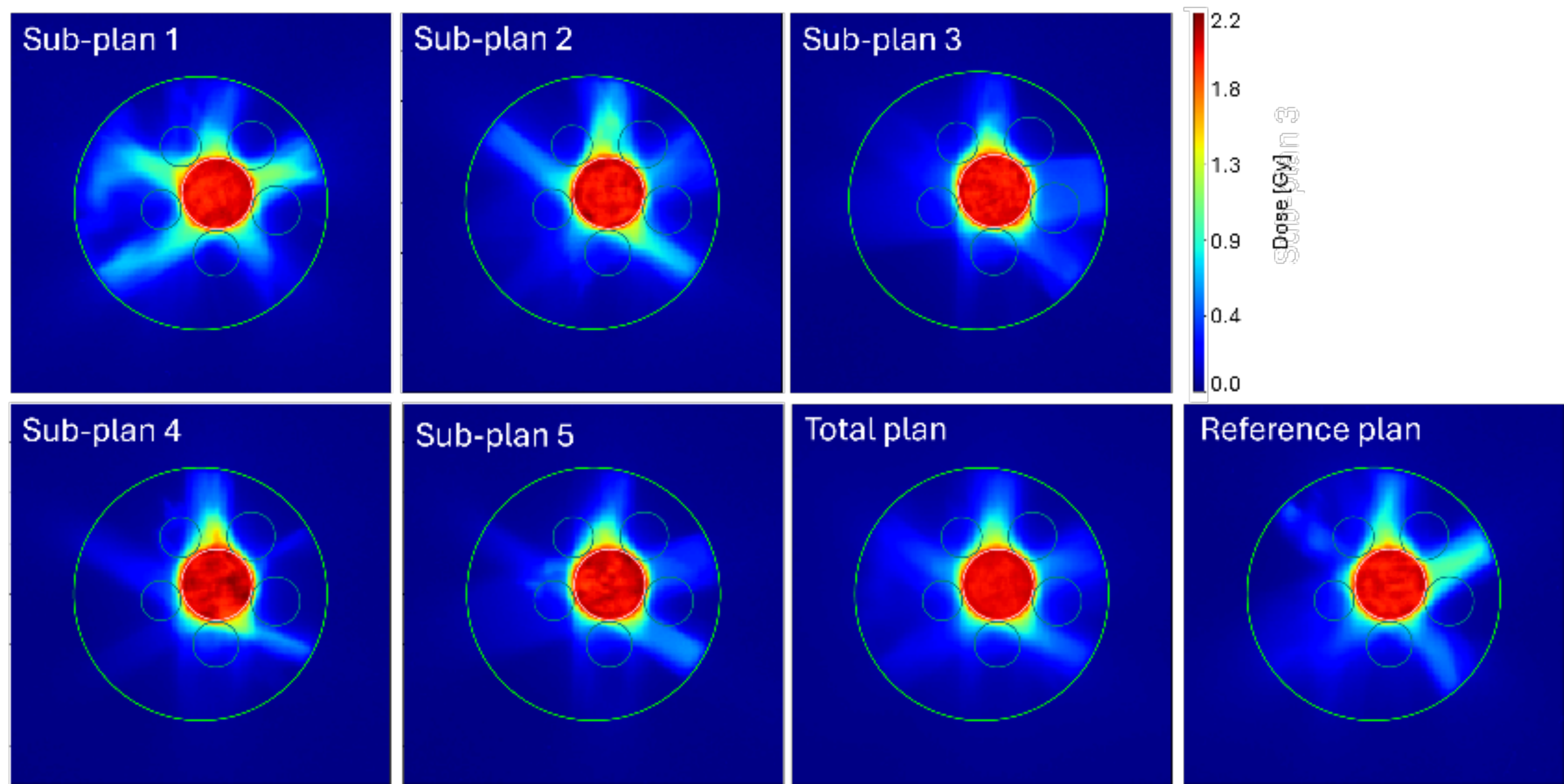

Supplement: Supplementary file 2 — Supporting information [file MP-52-0-s005.pdf]

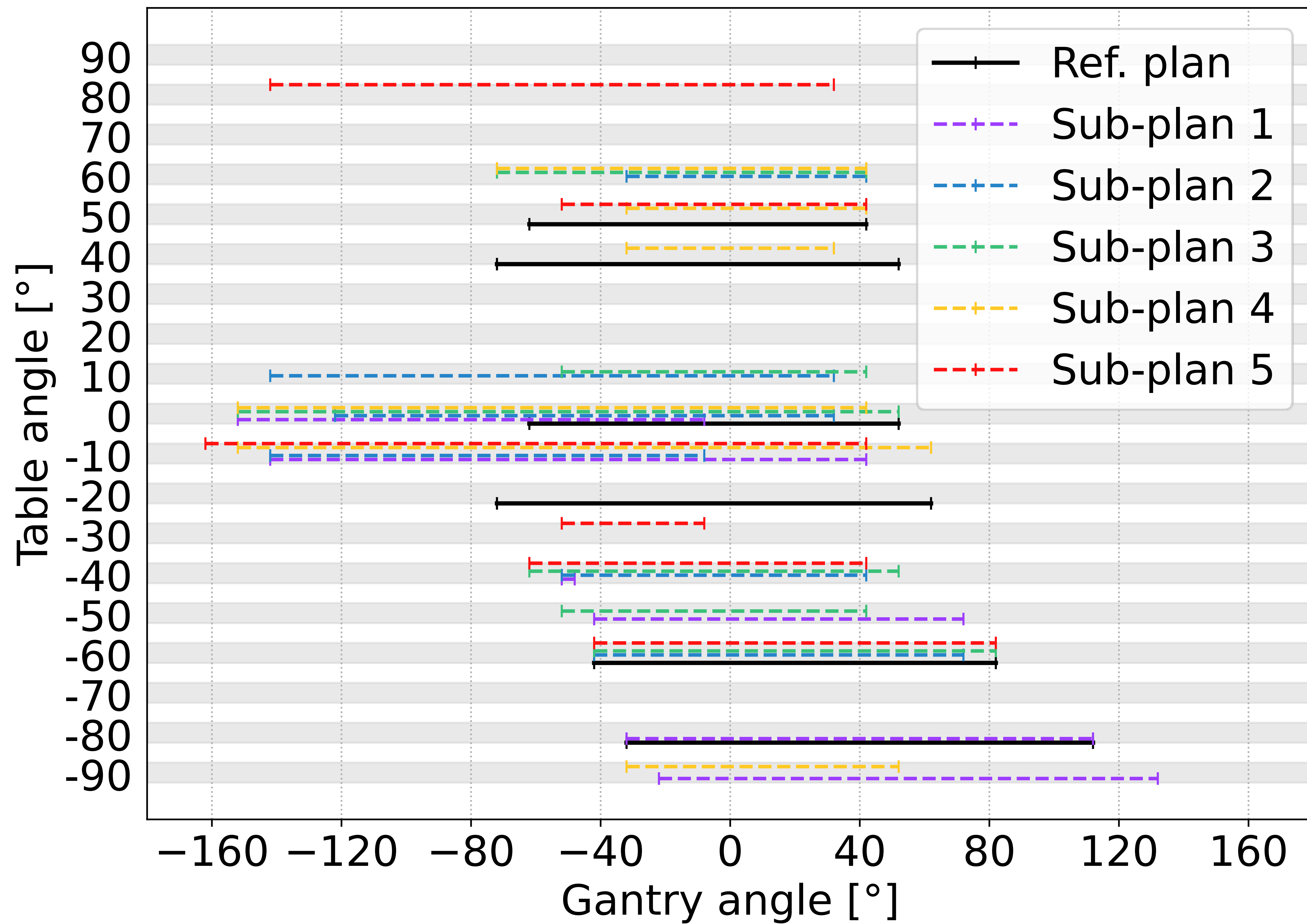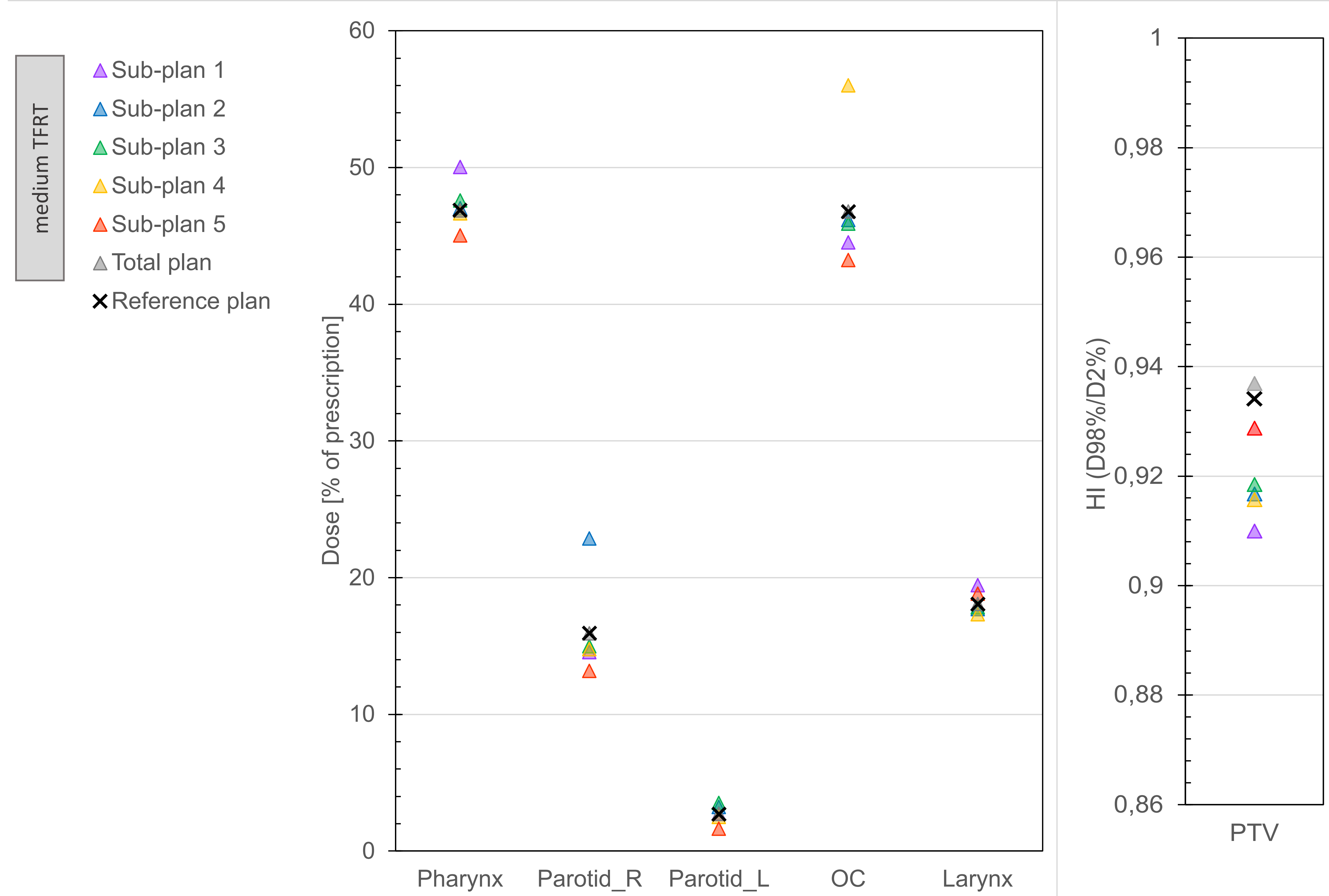

Supplement: Supplementary file 3 — Supporting information [file MP-52-0-s007.pdf]

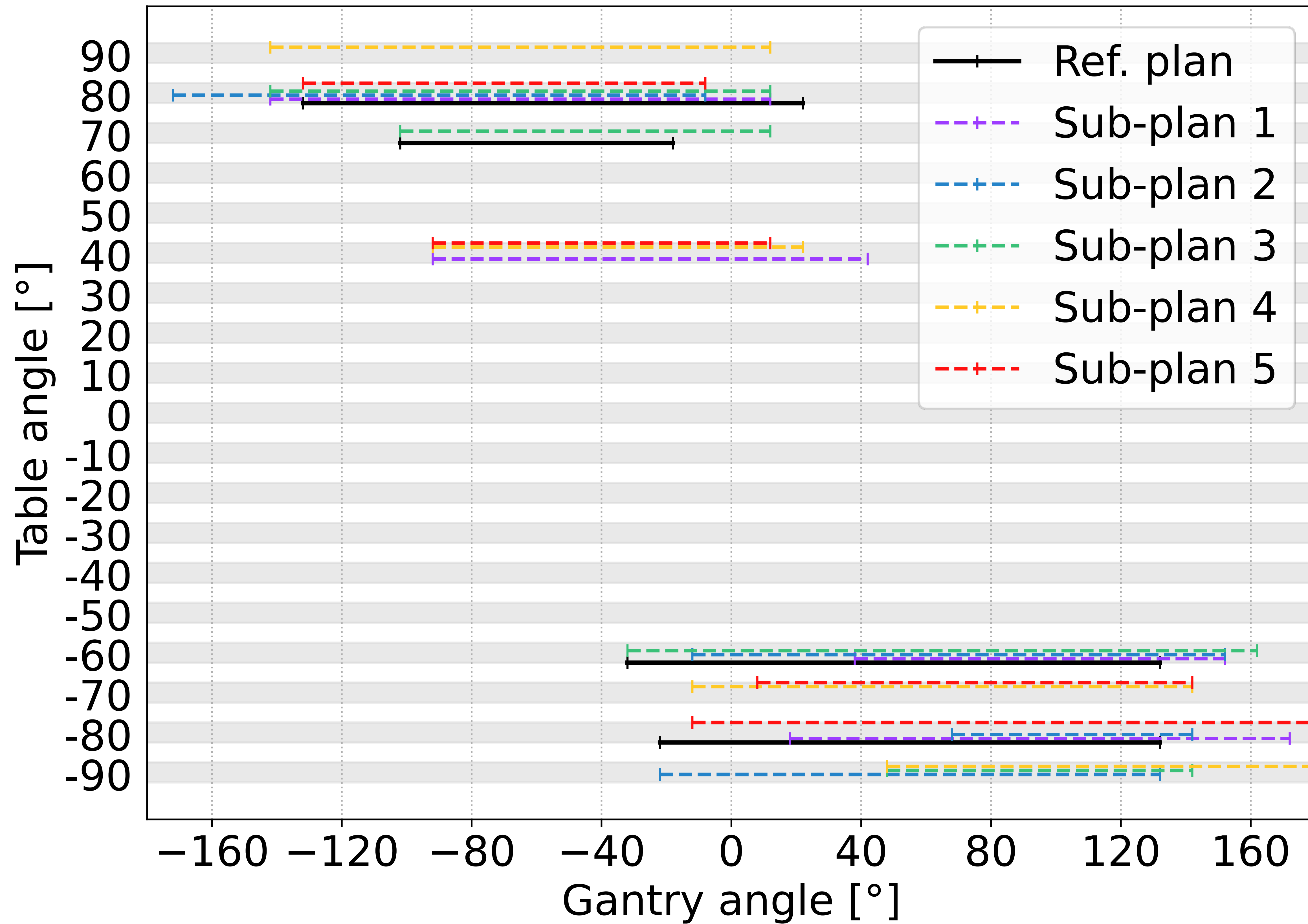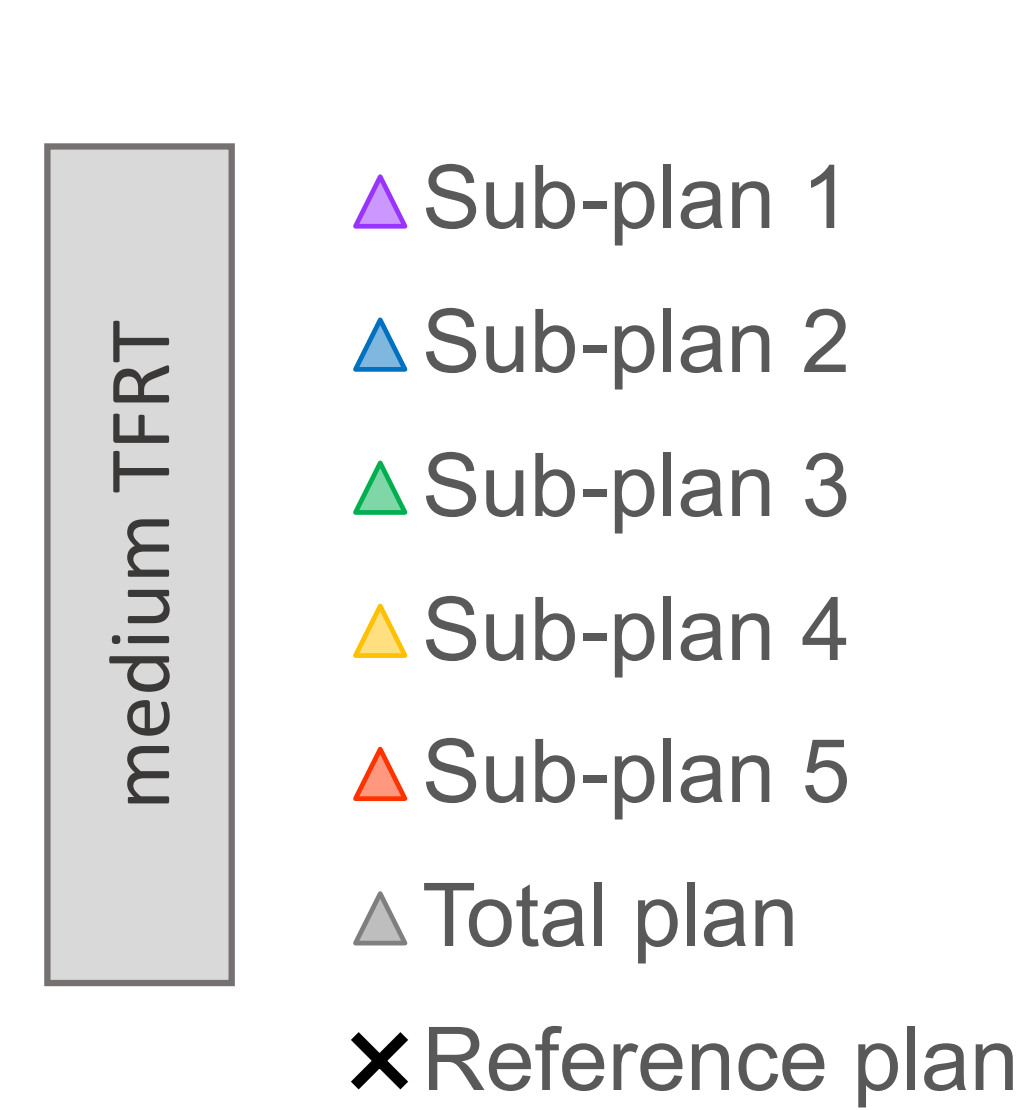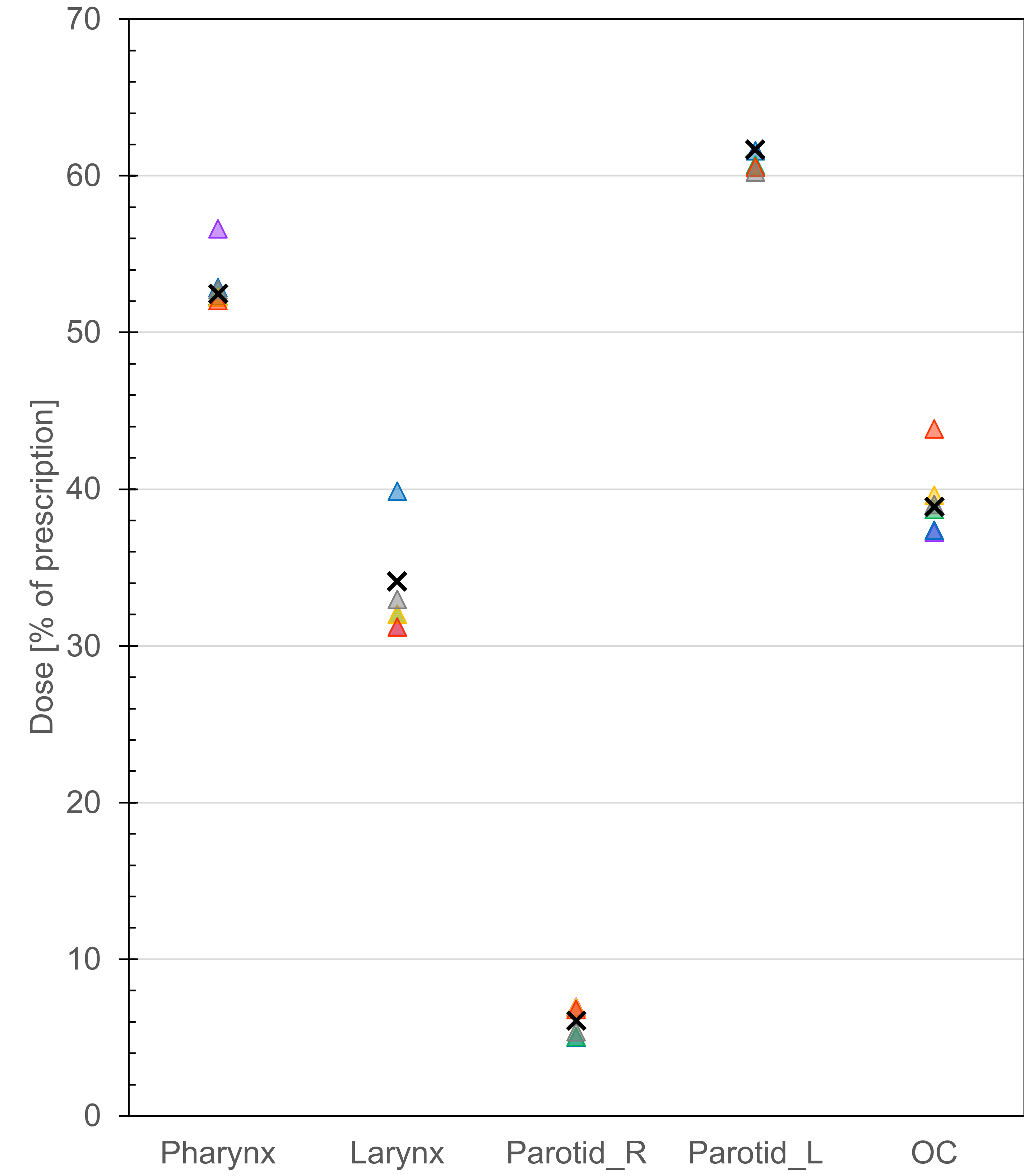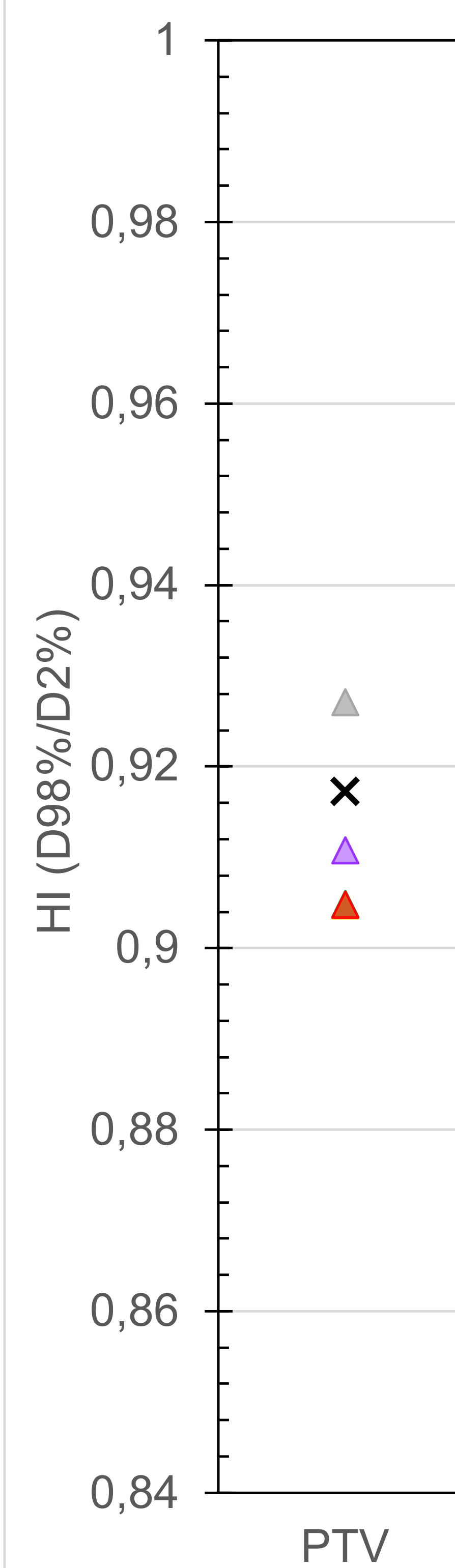

Supplement: Supplementary file 4 — Supporting information [file MP-52-0-s003.pdf]

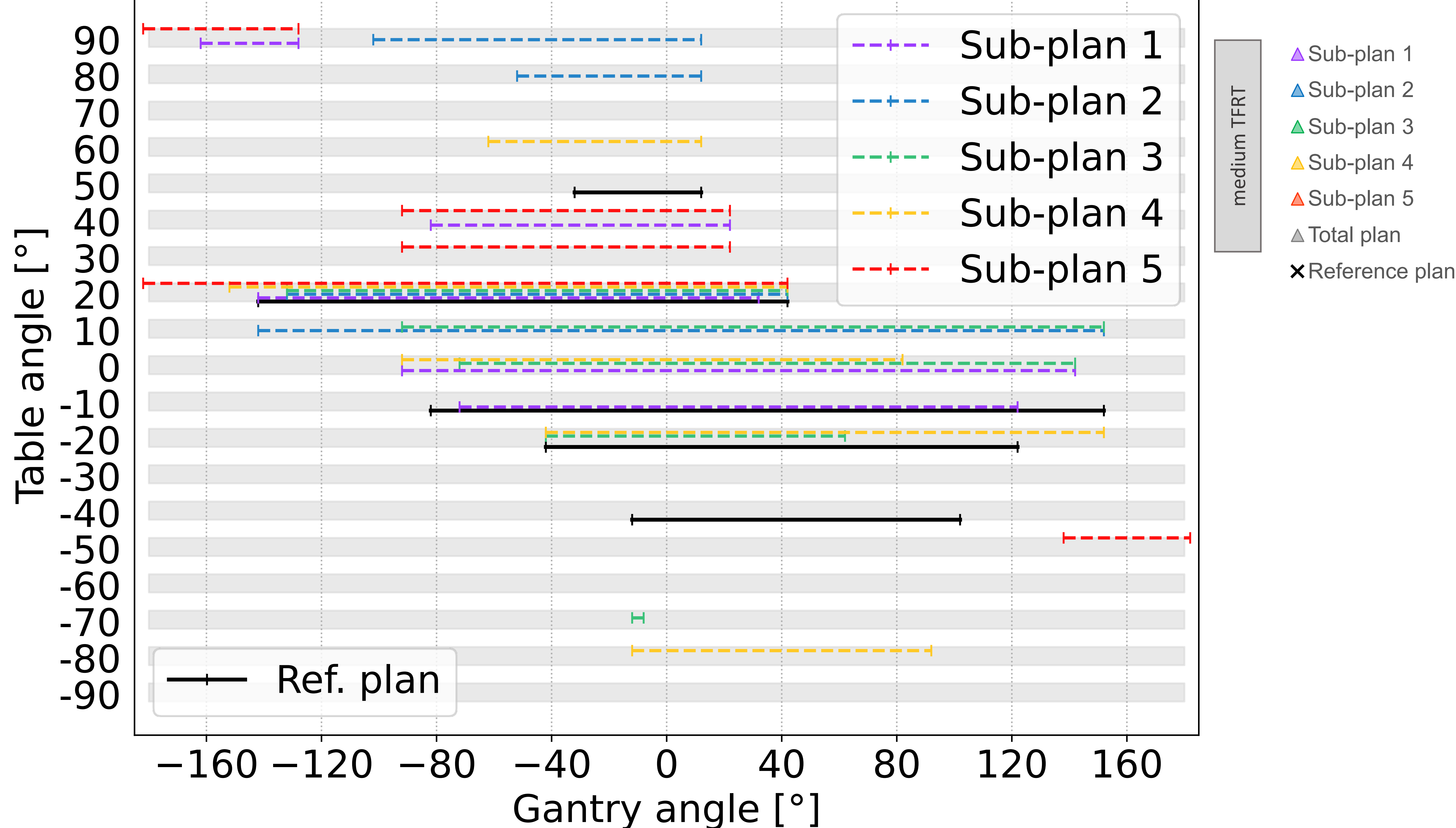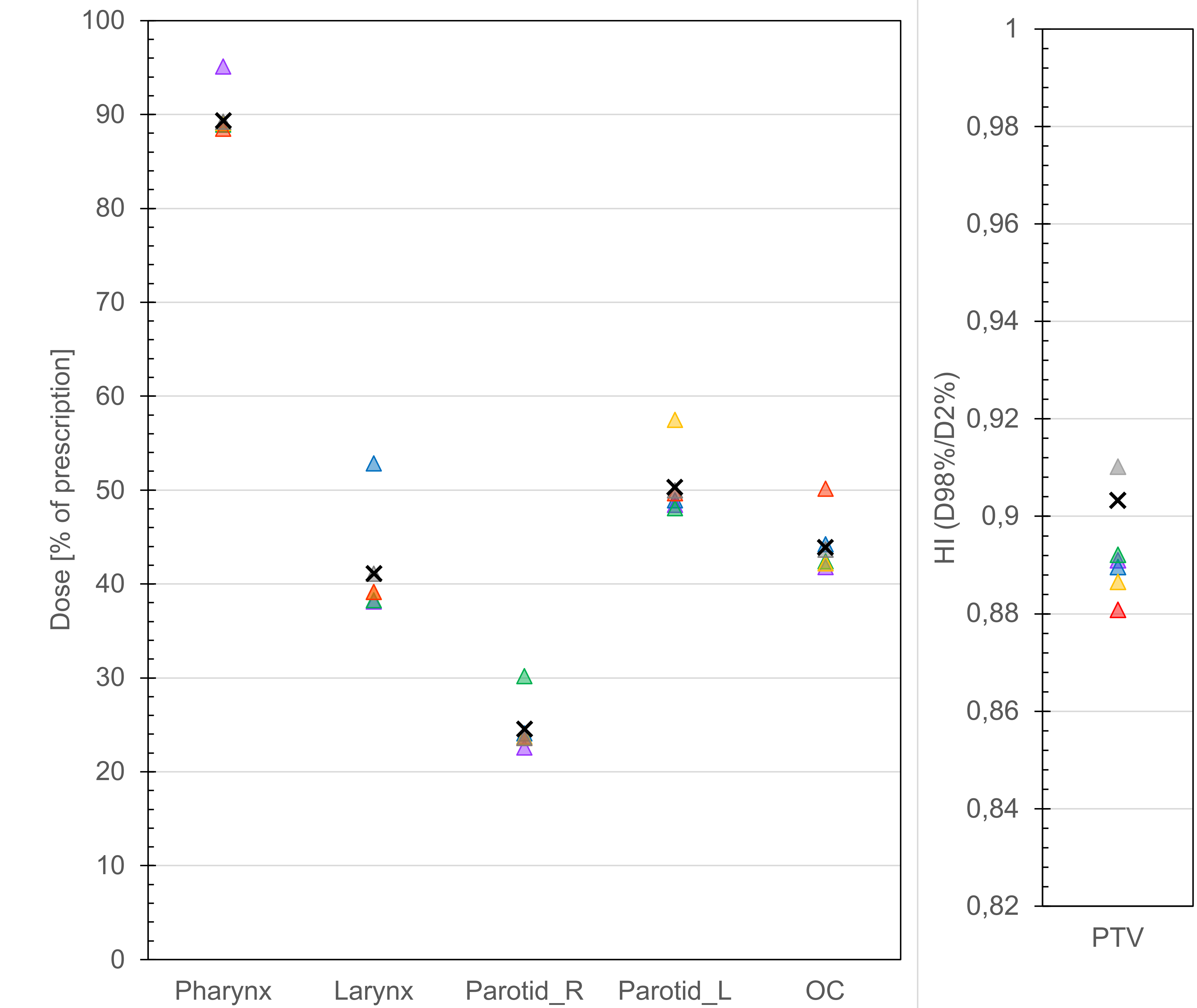

Supplement: Supplementary file 5 — Supporting information [file MP-52-0-s001.pdf]
