# Supplementary material for: Simultaneous optimization of multiple plans within one treatment course with dosimetric pathfinding for temporally feathered radiation therapy
Source: Med Phys. 2025 Sep 10;52(9):e18123. doi: 10.1002/mp.18123 (PMC12421372; doi:10.1002/mp.18123)
Supplement: Supplementary file 6 — Supporting information [file MP-52-0-s002.pdf]

Suppl. Table 1: Dose endpoints with clinical goals for non-feathered OARs. The best endpoints between the total plan and the reference plan are underlined. For sub-plans, the dose is scaled to a full course dose to facilitate comparison. PRV: Planning organ at risk volume.

Structure	Clinical goal	Case 1			Case 2			Case 3		
		Sub-plans (range)	Total TFRT	Ref	Sub-plans (range)	Total TFRT	Ref	Sub-plans (range)	Total TFRT	Ref
Mandatory										
Spinal cord	$D_{0.03\ cc} \leq$ 45 Gy	23.0 – 25.5	23.5	23.0	30.0 – 34.5	33.0	30.0	32.0 – 34.5	32.0	32.0
Spinal cord PRV	$D_{0.03\ cc} \leq$ 48 Gy	29.0 – 31.5	30.0	28.0	39.0 – 41.5	39.5	39.0	40.0 – 43.5	40.0	40.0
Brainstem	$D_{0.03\ cc} \leq$ 54 Gy	20.0 – 23.0	21.5	21.0	26.5 – 30.5	27.5	29.0	14.0 – 18.0	15.0	15.5
Brainstem PRV	$D_{0.03\ cc} \leq$ 54 Gy	22.0 – 26.5	25.0	23.0	31.5 – 35.0	32.5	33.0	21.0 – 24.0	22.0	23.0
ALARA										
Submandibular gland L	$D_{mean} \leq$ 35 Gy	2.0 – 2.0	2.0	2.0	43.0 – 43.5	43.0	44.0	35.0 – 36.5	35.0	35.0
Submandibular gland R	$D_{mean} \leq$ 35 Gy	Not contoured			5.0 – 6.5	5.5	7.0	31.0 – 32.0	31.5	31.0
Larynx GSL	$D_{mean} \leq$ 35 Gy	8.5 – 9.5	9.0	9.5	15.5 – 20.0	16.5	17.0	19.0 – 26.5	20.5	20.5
Esophagus	$D_{mean} \leq$ 40 Gy	Not contoured			18.0 – 19.0	18.5	18.0	19.0 - 19.5	19.0	19.0
Brachial Plexus	$D_{0.03cc} \leq$ 66 Gy	Not contoured			53.0 – 54.0	52.5	53.5	52.5 – 55.0	52.5	53.0
Mandible	$D_{2\%} \leq$ 70 Gy	47.5 – 49.0	47.5	47.0	51.0 – 53.5	51.0	52.0	48.0 – 51.0	48.0	49.0
Brain	$D_{0.03\ cc} \leq$ 65 Gy	27.0 – 33.0	31.0	31.0	43.5 – 44.5	43.0	44.0	27.0 – 36.5	32.0	31.0
Lips	$D_{mean} \leq$ 20 Gy	4.5 – 10.0	6.5	6.0	7.5 – 9.5	8.5	8.0	4.5 – 5.5	5.0	5.0
